# Solid cancer: the new tumour spread endpoint opens novel opportunities

**DOI:** 10.1038/s41416-019-0536-0

**Published:** 2019-08-20

**Authors:** Michael Fernandes, Daniel Rosel, Jan Brábek

**Affiliations:** 1 Medbase, 114 Milton Avenue, Chapel Hill, NC 27514 USA; 2 Department of Cell Biology, Faculty of Science and BIOCEV, Charles University, Prague, Czech Republic

**Keywords:** Metastasis, Cancer therapy

## Abstract

Novel androgen deprivation agents delay metastasis in non-metastatic, castration-resistant, prostate cancer (nmCRPC). The recent regulatory guidance: considerations for metastasis-free survival endpoint in clinical trials, opens new opportunities in cell biology, medicinal chemistry and advanced imaging. Past failures are the likely result of equating tumour shrinkage to efficacy, rather than inhibition of tumour spread. In the future, the selection of anti-metastasis drug candidates will probably be based on anti-migratory rather than anti-proliferative potential. Oligometastatic cancer coupled with advanced imaging can serve as a clinical proof-of-concept model.

## Main

The draft US Food and Drug Administration (FDA) guidance in solid cancer relative to a metastasis-free survival endpoint opens novel R&D opportunities towards meaningfully effective medicines.^1,2^ This guidance was prompted by the impressive and near-identical outcomes in three trials evaluating the efficacy of novel androgen deprivation therapies (ADTs) in non-metastatic, castration-resistant, prostate cancer (nmCRPC).^3–5^ Regulatory-mandated clinical trial endpoints are a powerful determinant of R&D direction and investment. The shift in focus from a tumour shrinkage paradigm towards a tumour spread format is long overdue.

nmCRPC is not a distinct and homogenous clinical entity, but a defined transition state between an indolent isolated cancer and an aggressive phase—metastatic CRPC (mCRPC). h-rnmCRPC is defined by rising levels (or a shorter doubling time) of prostate-specific antigen in the presence of castration levels of testosterone and the absence of radiographic evidence of distant metastases. About a third of patients develop a bone metastasis at 2 years; death from the disease is small and, in most cases, unrelated to the cancer. However, mCRPC is a progressive disease and associated with a poor prognosis despite conventional androgen deprivation therapy.

The trials on three novel ADTs were well-designed and well-conducted; the results were robust. On average, metastasis was delayed by 22 months, serious adverse events ranged between 5–8%, and discontinuations were few and balanced (Table 1). Prompted by the trial results, the FDA issued a draft guidance titled: ‘*Considerations for metastasis-free survival endpoint in clinical trials’*.^2^

**Table 1:** Novel androgen deprivation drugs. Summary data on three trials. (Modified from ref. ^3–5^)

	SPARTAN Jannsen		PROSPER Pfizer/Astellas		ARAMIS ORION/Bayer HealthCare	
	Apalutamide, Erleada		Enzalutamide, Extandi^®^		Darolutamide	
			
Group	A	P	E	P	D	P
Patients, n	806	421	933	468	955	554
M-Fs, m	40	18	37	15	40	18
SAE’s, %	25	20	31	23	25	20
Discontinuation, %	11	7	10	6	9	9

All subjects were on continuous conventional androgen deprivation therapy. Values are rounded

*A* apalutamide, *E* enzalutamide, *D*, darolutamide, *P* placebo, *M-FS* metastasis-free survival, months, *SAE’s* serious adverse events

### Regulatory-mandated clinical trial endpoints are a powerful determinant of R&D direction and investment

 The draft guidance reflects a shift in interest from tumour shrinkage to tumour spread. Drug-induced tumour shrinkage, which is easily measured, is rarely sustained and does not reflect a meaningful alteration in the natural history of solid cancer.^6^ To date, the approval of drugs for solid cancer are primarily based on tumour shrinkage. Progression-free survival may appear as a meaningful endpoint; it is not. Here, “progression” refers to an increase in tumour size not inhibition of metastasis—the main and proximate cause of morbidity and mortality. These considerations justify and rationalise the draft guidance.

The prevailing paradigm in clinical cancer research is that progressive disease is characterised by a parallel increase in tumour size and metastatic activity, and that drug-induced tumour shrinkage signifies inhibition of the metastatic process. Cell biologists explain the natural history of solid cancer as the result of two unrelated mechanisms: the increase in tumour size is a consequence of cell proliferation, and local invasion and subsequent metastasis results from migratory and invasive behaviour, as evidenced by the cancer cell traversing the basement membrane. This reasoning has led to the concept of “migrastatics”—agents that inhibit the directed motility of cells.^7^ In 1995, Ralph Weichselbaum and Samuel Hellman, proposed an intermediate state of metastases termed ‘oligometastases’.^8^ In this concept, the number and site of metastatic tumours are limited to 3–5 sites. An attractive consequence of this construct is that a just-activated metastatic process could be amenable to a curative strategy via drugs or stereotactic body radiation therapy.^9^ Reports continue to document better-than-expected outcomes for selected patients receiving radiotherapy for oligometastases.

### The ‘breakthrough’ from nmCRPC to metastatic CRPC suggests the entry of a new mechanism related to cancer cell motility

The development of resistance via a mutation in the androgen receptor could mechanistically relate to this “breakthrough” event. Specifically, the development of resistance of CRPC has been attributed to the enhanced expression of androgen receptors and its variants, notably AR-V7. Therefore, it is justifiable to explore preclinical screens based on the direct inhibition of cancer cell motility—the common denominator in invasive cancer. Here, leads are available from both synthetic biology and natural products, and especially from fragment-based ligand discovery coupled with 3D screening. Until now, the focus in discovery has been on cytotoxic agents; perhaps it is now time to direct efforts towards mechanistically-inspired anti-metastatic agents? The recent work at Cancer Research UK on anti-metastatic inhibitors of lysyl oxidase is an important step in this direction.^10^

In solid cancer, novel therapies require novel imaging approaches to identify earlier stages of the disease, the presence of metastases, and to detect successful responses.^10^ In this context, RECIST is not fit for purpose.^6,11^ Based on its ability to provide early detection of metastases, ^68^Ga-PSMA-11 PET imaging is appropriate for pharmacologic and radiotherapeutic interventional trials in oligometastatic prostate cancer.^12,13^ For the evaluation of candidate migrastatic agents we propose a trial in oligometastatic disease using advanced imaging technology and employing a common protocol. Continuing metastatic activity, however defined, is a meaningful endpoint; parallel measurements of tumour size would offer an excellent chance to compare the value of the two endpoints from the patients’ view. A small, careful and precise proof-of-concept trial in a selected population is best conducted at centres of excellence.^9^

In conclusion, we hope that partnerships between cell biology, medicinal chemistry, radiotherapy and imaging will catalyse a novel, different and rewarding approach to new drug discovery and development in most solid cancer. Trial success is the path towards valuable medicines that are prescribed for a longer duration, and at a lower price,^14^ and importantly help to answer the universal question of patients with solid cancer, ‘Doctor, has my cancer spread?’

## Data Availability

Not applicable.
